# Body Mass Index Peak-Rebound Patterns and Inhibitory Control Among Young Children

**DOI:** 10.1001/jamanetworkopen.2026.42834

**Published:** 2026-11-13

**Authors:** Jamil M. Lane, Izzuddin M. Aris, Hsiao-Hsien Leon Hsu, Emily Oken, Vishal Midya, Rosalind J. Wright, Daniel N. Klein, María Del Carmen Hernández-Chávez, María Luisa Pizano-Zárate, Martha M. Téllez-Rojo, Robert O. Wright

**Affiliations:** 1 Department of Environmental Medicine, Icahn School of Medicine at Mount Sinai, New York, New York; 2 Division of Chronic Disease Research Across the Lifecourse, Department of Population Medicine, Harvard Medical School and Harvard Pilgrim Health Care Institute, Boston, Massachusetts; 3 Department of Nutrition, Harvard T.H. Chan School of Public Health, Boston, Massachusetts; 4 Department of Pediatrics, Icahn School of Medicine at Mount Sinai, New York, New York; 5 Department of Public Health, Icahn School of Medicine at Mount Sinai, New York, New York; 6 Department of Psychology, Stony Brook University, Stony Brook, New York; 7 Department of Developmental Neurobiology, National Institute of Perinatology, Mexico City, Mexico; 8 Center for Nutrition and Health Research, National Institute of Public Health, Cuernavaca, Morelos, Mexico; 9 Institute for Exposomic Research, Icahn School of Medicine at Mount Sinai, New York, New York

## Abstract

**Importance:**

Childhood obesity has been linked with poor inhibitory control, and atypical body mass index (BMI) growth trajectories may identify children at higher risk for poor inhibitory control. Inhibitory control is also sexually dimorphic (male vs female), as maladaptive inhibitory control is more common in males.

**Objective:**

To investigate the association between early childhood BMI peak-rebound patterns and inhibitory control development at 4 to 5 and 6 to 7 years and test whether there is a sexually dimorphic association.

**Design, Setting, and Participants:**

The Programming Research in Obesity, Growth, Environment, and Social Stressors study is a prebirth cohort that recruited pregnant women receiving prenatal care through the Mexican Social Security Institute from July 2007 to February 2010 and followed up from pregnancy through age 8 years. Data were analyzed from November 2024 to December 2025.

**Exposure:**

Repeated weight and length or height measurements were collected from birth to 8 years and mixed-effects models were used to characterize sex- and child-specific BMI trajectories and derive age and magnitude of the BMI peak (maximum inflection point during infancy) and rebound (minimum inflection point during early childhood).

**Main Outcomes and Measures:**

Children completed the Conners Kiddie Continuous Performance Test, second edition, to assess inhibitory control at ages 4 to 5 and 6 to 7 years, and linear mixed-effects models were used to assess associations of age and magnitude of BMI peak and rebound with inhibitory control across the 2 time points. Sex-specific trajectories were evaluated by modeling interactions between child sex.

**Results:**

Among 539 children included in the study, 278 (51.6%) were male; 277 (51.4%) were born to mothers with low socioeconomic status, and the mean (SD) gestational age at birth was 38.4 (1.6) weeks. After adjusting for covariates, a 1-SD higher BMI at peak was associated with higher T-scores for hit reaction time standard errors (β = 0.89; 95% CI, 0.20 to 1.58). A 1-SD higher age at rebound was associated with higher hit reaction time (β = 2.35; 95% CI, −0.01 to 4.72). However, age at peak and BMI at rebound were not associated with any inhibitory control measure. Children with early timing of peak and rebound exhibited higher T-scores for omission errors (β = 4.05; 95% CI, 0.96 to 7.13), while children with early timing of peak and late timing of BMI rebound had lower T-scores for hit reaction time (β = −4.16; 95% CI, −7.29 to −1.02) compared with those with normal timing of peak and rebound. In males, both older age and a higher magnitude of BMI rebound were associated with lower omission error T-scores, whereas in females, variations in age and magnitude of BMI patterns were associated with higher T-scores for hit reaction time and hit reaction time standard errors.

**Conclusions and Relevance:**

In this cohort study of 539 children in Mexico, early-life BMI peak-rebound trajectories across childhood were associated with inhibitory control. These findings extend the literature beyond the well-established effects of BMI peak-rebound on metabolic conditions.

## Key Points

**Question:**

Are early-life BMI peak-rebound patterns across childhood associated with aspects of cognitive impairments, specifically in inhibitory control?

**Findings:**

In a prebirth cohort study of 539 Mexican children, variations in early childhood BMI trajectory milestones were associated with altered inhibitory control and attentional regulation, neurocognitive domains linked to impulsivity and inattentiveness. Among male children, variations in the timing and magnitude of BMI patterns were associated with more effective inhibitory control, whereas in female children, these BMI patterns were associated with less effective inhibitory control.

**Meaning:**

These findings suggest early changes in a child’s weight are associated with problems with attention and self-control later on.

## Teaser

This cohort study examines whether there is an association between early childhood body mass index peak-rebound patterns and inhibitory control development at ages 4 to 5 years and 6 to 7 years and, if so, whether the association varies by sex.

## Introduction

Childhood obesity, defined as a body mass index (BMI) in the 95th percentile or higher, has become a major global public health concern over the past 3 decades.^1,2^ In the US, childhood obesity affects nearly 15 million children; in Mexico, it affects 1 in 5 school-aged children,^3^ with nearly 40% of school-aged children (aged 5-11 years) living with obesity.^4,5^ Children who follow high-risk BMI growth patterns are more likely to develop obesity and a range of comorbid conditions as they age, including cardiovascular disease, psychological disorders, and several cancers.^6,7,8,9,10,11,12^ Understanding these BMI trajectory dynamics is therefore essential for effective prevention.

Substantial evidence has shown that obesity is intricately associated with prefrontal cortex impairments, leading to dysregulation in executive function (EF) development in children. EF is a meta-level construct representing several sophisticated cognitive and neurobehavioral processes that facilitate complex functions like attention, decision-making, emotional control, problem-solving, planning, and the ability to shift between attention and thought.^13,14,15,16^ EF development is essential during childhood and includes 3 domains: working memory, inhibitory control, and cognitive flexibility.^13,17^ Among EF domains, inhibitory control is fundamental, enabling humans to repress distractions and override behavioral and emotional responses.^18^ Inhibitory control is critical to children’s healthy development, supporting self-regulation, learning, academic performance, and adaptive behaviors, including eating behaviors throughout childhood. Some evidence suggests this relationship is bidirectional, with impairments in inhibitory control possibly preceding and contributing to later obesity by disrupting self-regulatory neurobehavioral processes, thereby emphasizing the role of impulsive eating behaviors.^19,20,21^ Behavioral studies have shown that maladaptive inhibitory control is more prevalent among children with higher BMI.^19,20,21,22,23^

Despite recognizing the neuroactive processes associated with excess adiposity, including neuroinflammation, metabolic, and hormonal signaling that may influence neural development and function,^24,25,26^ the intricate relationship between early-life BMI milestones, particularly BMI peak and rebound,^27,28^ and subsequent inhibitory control remains largely unknown. Prior research has demonstrated a link between the timing of these BMI milestones, such as later age at peak and earlier age at rebound, and later childhood obesity and cardiometabolic risk.^29,30,31^ However, we know of no studies that have examined how the timing and magnitude of BMI peak and rebound relate to the development of inhibitory control. Identifying early growth patterns associated with inhibitory control may help pinpoint sensitive developmental periods and inform future nutritional, behavioral, and healthy-growth strategies to support both cognitive and physical health. Furthermore, few studies have assessed sexually dimorphic effects (males vs females) of BMI peak-rebound patterns and inhibitory control.^32,33^ To address these gaps, we used a prospective prebirth cohort with repeated BMI measures and early school-age inhibitory control assessments to investigate the association between early childhood BMI peak-rebound trajectories and inhibitory control performance, and to explore whether this outcome is sexually dimorphic.

## Methods

### Study Cohort

The Programming Research in Obesity, Growth, Environment, and Social Stressors (PROGRESS) study is a prebirth cohort that recruited pregnant women receiving prenatal care through the Mexican Social Security Institute from July 2007 to February 2010. Full details of the cohort’s profile and enrollment are published elsewhere.^34,35^ Briefly, the eligibility criteria included being a singleton pregnant woman over 18 years of age, at less than 20 weeks of gestation, who planned to stay in Mexico City for the next 3 years. Other criteria consisted of access to a telephone, no major disease in medical history, and not using steroid or antiepilepsy medications or consuming alcohol daily. Institutional review boards at the Harvard School of Public Health, Icahn School of Medicine at Mount Sinai, the Mexican National Institute of Public Health, and the National Institute of Perinatology approved the recruitment procedures. All participants provided written informed consent at enrollment and during the follow-up visits. This study followed the Strengthening the Reporting of Observational Studies in Epidemiology (STROBE) reporting guideline for cohort studies.

We enrolled women in the early second trimester of pregnancy who gave birth to a live child who was then followed longitudinally. We based gestational age on the last menstrual period and standardized physical examination.^36^ Mother-child dyads returned for study visits every 2 years. The current analysis included children who completed the inhibitory control assessment at ages 4 to 5 and 6 to 7 years and had 2 or more BMI measures from birth to 8 years.

### Body Mass Index

Trained staff collected weight and length measures every 6 months from birth to 2 years, and weight and standing height using a professional digital scale (Health-o-meter) at ages 2, 4, 6, and 8 years. We calculated BMI as weight in kilograms divided by the square of length or height in meters.

### Inhibitory Control Assessment

At the 4 to 5– and 6 to 7–year visits, trained staff administered the Conners Kiddie Continuous Performance Test second Edition (K-CPT), a well-established, validated tool for evaluating inattentiveness, impulsiveness, sustained attention, and vigilance through the presentation of visual cues and the measurement of appropriate responses.^37^ The K-CPT tasks included omission errors (OE), commission errors (CE), hit reaction time (HRT) and its standard error (HRT SE), perseveration responses (PR), and response style (RS). We selected the 4 to 5–year and 6 to 7–year assessments because they corresponded to the developmental age range for which the K-CPT was designed and provided repeated assessments of inhibitory control during early childhood. Table 1 describes each K-CPT neurobehavioral variable used as a primary outcome in the current study. The eMethods in Supplement 1 provide detailed descriptions of the KCPT protocol, administration, and scoring procedures.

**Table 1.** Description of Each Conners Kiddie Continuous Performance Test Task

Measures	Description of tasks	Dimensions
Omission errors	Failing to respond to a target stimulus	Inattentiveness
Commission errors	Responding to a nontarget stimulus	Inattentiveness and impulsivity
Hit reaction time	Speed of correct responses to target stimuli (response speed)	Inattentiveness and impulsivity
Hit reaction time standard error	Consistency or variability in correct response times over the whole test	Inattentiveness
Perseveration responses	Difficulty shifting responses; repeating the same response despite changes in stimuli	Impulsivity
Response style	Overall responding tendencies (eg, overly cautious or impulsive)	Impulsivity

### Statistical Analysis

We conducted the main analyses in 2 stages. In stage 1, we modeled child-specific BMI curves to estimate BMI milestones (age and magnitude at BMI peak and rebound) for each child. In stage 2, we examined associations of each BMI milestone with inhibitory control outcomes. Prior work has shown that this approach provides unbiased estimates of the associations between growth parameters and outcomes.^38^

We used statistical models to estimate the age and magnitude of BMI peak and rebound, which have been shown to provide more precise estimates of these milestones,^39^ rather than direct observations and visual inspection of individual BMI-for-age curves, which are prone to large interobserver variation and measurement error. In line with prior work,^39,40^ we fitted child-specific BMI curves from birth to 8 years using mixed-effects models with natural cubic spline functions for age to capture BMI nonlinearity. The most optimal model (ie, lowest bayesian information criterion value) included 3 internal knots placed at ages 0.5, 1, and 4 years and 2 boundary knots at ages 0 and 8 years. We included interactions of child sex with spline terms as fixed effects, along with random effects for the intercept, linear age slopes, and spline functions to account for repeated BMI measures within each child. Using the fitted model, we estimated the exact age (in years) at peak in infancy and rebound in childhood by differentiating each child-specific BMI curve and locating the ages where the derivative of the curve equals 0 (ie, at the maximum inflection point during infancy for peak, and the minimum inflection point during childhood for rebound). We estimated the magnitude of BMI at peak and rebound as the highest and lowest points, respectively, of each child-specific BMI curve. Although we used BMI from birth to 8 years to derive child-specific BMI curves, our exposure of interest pertains only to BMI peak in infancy and rebound in childhood, which are point measures on the BMI curve that occur at or before the outcome.

We used linear mixed-effects models to assess associations of each estimated BMI milestone with inhibitory control outcomes across ages 4 to 5 and 6 to 7 years. We modeled each inhibitory control outcome separately, expressed per 1-SD increase in each BMI milestone, to allow direct comparisons across different BMI milestones. Model 1 was not adjusted for covariates. Model 2 adjusted for the socioeconomic status index, birth weight, gestational age at delivery, secondhand smoke exposure, maternal BMI, and maternal IQ. All models included a random intercept to account for repeated inhibitory control measures. In line with prior work,^40^ we included both age and magnitude (at peak or rebound) in the same model to disentangle each milestone’s independent association with the outcome. For models with age and magnitude at rebound as the variables, we additionally adjusted for age and magnitude at peak (which occurs before BMI rebound) but did not adjust models of BMI peak for characteristics of BMI rebound, which occurs later. We conducted exploratory sex-specific analyses by including interactions of BMI milestones with child sex in the regression models. Additionally, as reference ranges for age at peak or rebound have not been established, we categorized each as early (<25th percentile), normal (≤25th to ≥75th percentile), or late (>75th percentile) internally within the cohort in accordance with prior work,^40^ resulting in 9 categories of relative timing of BMI peak and rebound. We assessed associations of each peak-rebound category (vs normal timing of BMI peak and rebound) with each inhibitory control outcome, adjusting for the same covariates listed above. eMethods in Supplement 1 present additional information on the covariates included in the analyses. We performed all analyses in R version 4.3 (R Project for Statistical Computing) with 2-sided α < .05 to assess statistical significance, although interpretation focused on the direction, strength, and precision of the estimates.^41^ Data were analyzed from November 2024 to December 2025.

## Results

### Cohort Characteristics

Table 2 describes the characteristics of children in the analytic sample with an estimable BMI peak and rebound. Among 539 children included in the study, 278 (51.6%) were male; 277 (51.4%) were born to mothers with low socioeconomic status, and the mean (SD) gestational age at birth was 38.4 (1.6) weeks. The mean (SD) age at peak was 1.0 (0.3) years and at rebound was 4.9 (1.0) years, and the mean (SD) magnitude of BMI at peak was 17.0 (1.2) and at rebound was 15.4 (1.2). Female children had a later age at peak and a lower BMI at peak and rebound than male children. Mean age-standardized T-scores of sustained attention and impulsivity were all close to the standardized population values (mean [SD], 50 [10]).

**Table 2.** Sample Characteristics of Mother-Child Dyads in the Study Cohort

Characteristic	Dyads, No.	Overall sample, No. (%)	Males (n = 278)	Females (n = 261)
BMI milestones^a^				
Age at BMI peak, mean (SD), y	539	1.0 (0.3)	0.9 (0.2)	1.0 (0.3)
BMI at peak, mean (SD)	539	17.0 (1.2)	17.2 (1.1)	16.9 (1.3)
Age at BMI rebound, mean (SD), y	539	4.9 (1.0)	4.9 (0.9)	4.9 (1.0)
BMI at rebound^, ^mean (SD)	539	15.4 (1.2)	15.4 (1.1)	15.4 (1.3)
BMI peak-rebound patterns				
Normal AP plus normal AR	539	135 (25.0)	72 (25.9%)	63 (24.1%)
Early AP plus early AR	539	38 (7.1)	23 (8.3%)	15 (5.7%)
Early AP plus normal AR	539	44 (8.2)	27 (9.7%)	17 (6.5%)
Early AP plus late AR	539	47 (8.7)	27 (9.7%)	20 (7.7%)
Normal AP plus early AR	539	71 (13.2)	39 (14.0%)	32 (12.3%)
Normal AP plus late AR	539	97 (18.0)	47 (16.9%)	50 (19.2%)
Late AP plus early AR	539	18 (3.3)	6 (2.2%)	12 (4.6%)
Late AP plus normal AR	539	61 (11.3)	24 (8.6%)	37 (14.2%)
Late AP plus late AR	539	28 (5.2)	13 (4.7%)	15 (5.7%)
Maternal and child characteristics				
Maternal SES				
Low	539	277 (51.4)	141 (50.7%)	136 (52.1%)
Medium	539	210 (39.0)	111 (39.9%)	99 (37.9%)
High	539	52 (9.6)	26 (9.4%)	26 (10.0%)
Secondhand smoke at second trimester	539			
No		375 (69.6)	197 (70.9%)	178 (68.2%)
Yes		164 (30.4)	81 (29.1%)	83 (31.8%)
Gestational age at birth, mean (SD), wk	539	38.4 (1.6)	38.2 (1.7)	38.5 (1.6)
Maternal BMI at second trimester, mean (SD)	539	26.7 (4.1)	27.1 (4.0)	26.3 (4.2)
Maternal IQ, mean (SD)	539	84.9 (12.4)	84.8 (13.0)	85.0 (11.8)
Birth weight, mean (SD), kg	539	3.1 (0.4)	3.1 (0.4)	3.0 (0.4)
Child age at age 4-5 y visit, mean (SD), y	538	4.8 (0.5)	4.8 (0.5)	4.8 (0.5)
Child age at age 6-7 y visit, mean (SD), y	507	6.7 (0.6)	6.7 (0.6)	6.7 (0.5)
Inhibitory control measures, mean (SD)				
Commission error T-scores at age 4-5 y	538	56.2 (9.5)	56.1 (8.9)	56.4 (10.1)
Commission error T-scores at age 6-7 y	105	49.7 (9.0)	50.9 (8.1)	48.7 (9.6)
HRT T-scores at age 4-5 y	539	49.3 (9.4)	49.2 (9.1)	49.5 (9.8)
HRT T-scores at age 6-7 y	105	52.6 (10.7)	52.6 (10.1)	52.6 (11.2)
HRT SE T-scores at age 4-5 y	538	52.6 (7.6)	53.1 (7.0)	52.2 (8.2)
HRT SE T-scores at age 6-7 y	105	54.5 (9.7)	54.3 (9.4)	54.7 (10.1)
Omission error T-scores at age 4-5 y	539	49.1 (8.8)	48.0 (8.8)	50.2 (8.7)
Omission error T-scores at age 6-7 y	105	55.4 (10.8)	55.7 (11.3)	55.2 (10.5)
Perseverations T-scores at age 4-5 y	539	59.2 (18.4)	60.1 (18.1)	58.3 (18.6)
Perseverations T-scores at age 6-7 y	105	54.0 (15.3)	53.3 (15.0)	54.6 (15.8)
Response style T-scores at age 4-5 y	539	49.3 (11.2)	48.9 (9.7)	49.8 (12.6)
Response style T-scores at age 6-7 y	105	50.6 (6.4)	51.8 (8.8)	49.5 (2.6)

Abbreviations: AP, age at peak; AR, age at rebound; BMI, body mass index; HRT, hit reaction time; HRT SE, hit reaction time standard error; SES, socioeconomic status.

^a^ Calculated as weight in kilograms divided by height in meters squared.

### Association Between BMI Milestones and Inhibitory Control Outcomes

After adjusting for covariates, a 1-SD unit increment in age at BMI peak was not associated with any of the attention or impulsivity measures across assessments at ages 4 and 6 years, including CE T-scores (β = 0.60; 95% CI, −0.61 to 1.81) and OE T-scores (β = −0.78; 95% CI, −1.91 to 0.34) (Table 3). A 1-SD unit higher in magnitude of BMI at peak was associated with higher T-scores for HRT SE (β = 0.89; 95% CI, 0.22 to 1.54). A 1-SD unit higher age at BMI rebound was associated with higher HRT (β = 2.35; 95% CI, 0.04 to 4.66). However, magnitude of BMI at rebound was not associated with any of the attention and impulsivity measures.

**Table 3.** Associations of Ages at Body Mass Index (BMI) Peak and Rebound, and Magnitudes of BMI at Peak and Rebound With Conners Kiddie Continuous Performance Test (K-CPT) T-Scores^a^

Variable	Commission error T-scores (n = 537)^b^		HRT T-scores (n = 538)^c^		HRT SE T-Scores (n = 537)^c^		Omission error T-scores (n = 538)^b^		Perseverations T-scores (n = 538)		Response style T-scores (n = 538)	
	Model 1^d^	Model 2^e^	Model 1^d^	Model 2^e^	Model 1^d^	Model 2^e^	Model 1^d^	Model 2^e^	Model 1^d^	Model 2^e^	Model 1^d^	Model 2^e^
Age at BMI peak	0.45 (−0.73 to 1.63)	0.59 (−0.60 to 1.79)	−0.12 (−1.36 to 1.12)	−0.17 (−1.43 to 1.08)	0.05 (−0.95 to 1.05)	−0.02 (−1.03 to 0.99)	−0.43 (−1.54 to 0.69)	−0.54 (−1.67 to 0.58)	0.24 (−2.03 to 2.52)	0.20 (−2.10 to 2.51)	−0.48 (−1.78 to 0.82)	−0.50 (−1.83 to 0.83)
BMI at peak	0.64 (−0.15 to 1.42)	0.81 (−0.01 to 1.62)	0.30 (−0.52 to 1.12)	0.40 (−0.45 to 1.25)	0.87 (0.21 to 1.54)	0.90 (0.22 to 1.59)	0.48 (−0.26 to 1.22)	0.50 (−0.26 to 1.27)	1.07 (−0.44 to 2.57)	0.98 (−0.59 to 2.54)	−0.57 (−1.44 to 0.29)	−0.55 (−1.46 to 0.35)
Age at BMI rebound^f^	−0.40 (−2.55 to 1.75)	−0.79 (−2.99 to 1.41)	2.17 (−0.09 to 4.42)	2.35 (0.04 to 4.66)	−0.16 (−1.98 to 1.66)	−0.13 (−1.98 to 1.73)	−1.19 (−3.21 to 0.82)	−0.92 (−2.98 to 1.13)	−1.47 (−5.60 to 2.67)	−1.15 (−5.39 to 3.10)	−0.68 (−3.03 to 1.67)	−0.60 (−3.03 to 1.82)
BMI at rebound^f^	−0.64 (−3.47 to 2.20)	−1.03 (−3.91 to 1.85)	2.06 (−0.92 to 5.03)	2.11 (−0.92 to 5.13)	−0.37 (−2.77 to 2.04)	−0.51 (−2.93 to 1.92)	−0.78 (−3.44 to 1.89)	−0.77 (−3.47 to 1.93)	−1.68 (−7.14 to 3.79)	−1.34 (−6.90 to 4.21)	−0.44 (−3.56 to 2.67)	−0.13 (−3.31 to 3.06)

Abbreviations: HRT, hit reaction time; SE, standard error.

^a^ Effect estimates and 95% CIs represent mean differences in K-CPT T-scores per 1 SD increment in age and magnitude of BMI at peak and/or rebound.

^b^ Higher scores reflect higher error rates, corresponding to reduced task performance.

^c^ Higher scores reflect slow reaction times (HRT) and greater variability (HRT SE), respectively, both of which indicate inattentiveness.

^d^ Unadjusted model.

^e^ Adjusted for socioeconomic status index, birth weight, gestational age at delivery, secondhand smoke exposure, maternal BMI, and maternal intelligence quotient.

^f^ Additionally adjusted for both age and magnitude of BMI at peak.

Furthermore, children with early timing of BMI peak and rebound exhibited higher T-scores for OE (β = 4.05; 95% CI, 0.96 to 7.13), while children with early timing of BMI peak and late timing of BMI rebound had lower T-scores for HRT (β = −4.16; 95% CI, −7.29 to −1.02) compared with those with normal timing of BMI peak and rebound (Table 4).

**Table 4.** Association Between Timing of Body Mass Index (BMI) Peak-Rebound Patterns in Categories and Conners Kiddie Continuous Performance (K-CPT) T-Scores^a^

Variable	T-score outcomes, β (95% CI)					
	Commission errors	HRT	HRT SE	Omission errors	Perseverations	Response style
Normal AP plus normal AR	[Reference]	[Reference]	[Reference]	[Reference]	[Reference]	[Reference]
Early AP plus early AR	−0.88 (−4.19 to 2.42)	−0.85 (−4.27 to 2.57)	1.78 (−1.00 to 4.56)	4.05 (0.96 to 7.13)	1.69 (−4.63 to 8.01)	1.24 (−2.41 to 4.90)
Early AP plus normal AR	−0.28 (−3.36 to 2.81)	−1.50 (−4.70 to 1.69)	−0.83 (−3.43 to 1.77)	−0.27 (−3.15 to 2.62)	−1.72 (−7.63 to 4.18)	−0.76 (−4.17 to 2.65)
Early AP plus late AR	1.12 (−1.91 to 4.15)	−4.16 (−7.29 to −1.02)	−0.56 (−3.11 to 1.99)	−0.11 (−2.94 to 2.71)	1.30 (−4.50 to 7.10)	−2.33 (−5.67 to 1.02)
Normal AP plus early AR	−0.18 (−2.85 to 2.49)	−2.07 (−4.83 to 0.68)	−1.06 (−3.31 to 1.18)	0.11 (−2.39 to 2.61)	−0.21 (−5.31 to 4.90)	0.01 (−2.95 to 2.97)
Normal AP plus late AR	−0.28 (−2.62 to 2.06)	0.63 (−1.80 to 3.06)	0.47 (−1.51 to 2.44)	0.48 (−1.70 to 2.66)	0.40 (−4.08 to 4.88)	−0.51 (−3.09 to 2.07)
Late AP plus early AR	−0.92 (−5.45 to 3.61)	−1.82 (−6.50 to 2.85)	−1.16 (−4.98 to 2.65)	0.45 (−3.80 to 4.70)	−4.25 (−12.92 to 4.41)	−2.86 (−7.90 to 2.17)
Late AP plus normal AR	0.47 (−2.27 to 3.22)	−2.16 (−4.99 to 0.68)	−0.36 (−2.66 to 1.93)	−0.88 (−3.41 to 1.65)	4.55 (−0.67 to 9.77)	−1.64 (−4.63 to 1.35)
Late AP plus late AR	−0.05 (−3.74 to 3.64)	1.82 (−2.00 to 5.64)	1.20 (−1.90 to 4.31)	1.69 (−1.76 to 5.14)	−2.38 (−9.45 to 4.68)	0.56 (−3.52 to 4.64)

Abbreviations: AP, age at BMI peak; AR, age at BMI rebound; HRT, hit reaction time; HRT SE, hit reaction time standard error.

^a^ Early AP or AR indicates age 25th percentile or less; normal AP or AR, age greater than 25th or 75th percentile or less; late AP or AR, age greater than 75th percentile. All models adjusted for socioeconomic status index, birth weight, gestational age at delivery, secondhand smoke exposure, maternal BMI, and maternal intelligence quotient.

### Sex-Specific Associations Between BMI Milestones and Inhibitory Control Outcomes

Our exploratory analyses suggest sexually dimorphic associations of age at BMI peak (*P* for interaction = .04), age at BMI rebound (*P* for interaction = .15), and magnitude of BMI rebound (*P* for interaction = .08) with OE T-scores and of age at BMI rebound with HRT T-scores (*P* for interaction = .10) (eTable 1 in Supplement 1). Specifically, in male children, a 1-SD unit higher age at BMI peak (β = −2.55; 95% CI, −4.42 to −0.68) as well as higher age (β = −3.20; 95% CI, −6.31 to −0.09) and magnitude of BMI at rebound (β = −4.30; 95% CI, −8.24 to −0.37) were associated with lower OE T-scores. However, neither age nor BMI at peak was associated with any of the attention and impulsivity measures in male children (eTable 1 in Supplement 1). Male children with early age at peak and rebound exhibited higher T-scores for PR (β = 9.51; 95% CI, 0.99 to 18.03). Male children with early age at peak and normal age at rebound had lower OE T-scores, and those with early age at peak and late age at rebound had higher CE T-scores and lower HRT T-scores (eTable 2 in Supplement 1). Those with normal age at peak and early age at rebound had lower RS T-scores, while those with late age at peak and early age at rebound had lower T-scores for HRT SE and OE (eTable 2 in Supplement 1).

In female children, a 1-SD unit higher age at BMI rebound was associated with higher HRT T-scores (β = 4.49; 95% CI, 1.07 to 7.90). However, age at peak as well as magnitude of BMI at peak and rebound were not associated with any of the attention and impulsivity measures (eTable 1 in Supplement 1). Female children with early age at peak and rebound exhibited lower CE T-scores and higher T-scores for OE and RS, while those with early age at peak and normal timing of rebound had higher OE T-scores (eTable 3 in Supplement 1).

## Discussion

In this Mexican cohort, age and magnitude at BMI peak and rebound showed associations with school-age K-CPT performance, reflecting patterns consistent with differences in inhibitory control and attentional regulation, neurocognitive domains related to impulsivity and inattentiveness. However, given the number of comparisons examined, these findings should be considered exploratory. Higher BMI at peak was associated with a greater frequency of CE, suggesting poorer inhibitory control and greater variability in response speed across the test (higher HRT SE), a marker of attentional inconsistency. Older age at BMI rebound was associated with higher HRT (longer reaction times), suggesting attentional inefficiency and slower cognitive speed. Interestingly, children with earlier timing of both milestones exhibited greater frequency of OE than children with normative timing for both BMI milestones. In contrast, an early BMI peak and late rebound showed lower response-speed variability. This pattern may be compatible with impulsive responding without accompanying fluctuations in attentional efficiency and lapses in processing consistency.

To our knowledge, no study to date has examined how BMI peak-rebound patterns are associated with children’s inhibitory control and attentional regulation. Elevated adiposity in childhood has been associated with both the structural and functional integrity of brain regions, including prefrontal and limbic regions,^42,43^ particularly during the early years of life. These regions may represent susceptible neuroanatomical correlates of childhood adiposity, with observed differences characterized by heightened reward sensitivity and diminished interoceptive processing, particularly during food-related decision-making.^44^ Longitudinal evidence shows that higher BMI may be linked to subsequent changes in brain morphology; however, the directionality of these relationships remains complex.^43,45^ Recent evidence supports bidirectional associations, whereby EF and prefrontal morphology may contribute to and be influenced by subsequent adiposity.^45^ Common genetic influences may independently shape both adiposity and neurocognitive development, providing an alternative explanation for their observed associations beyond a direct causal relationship.

Patterns of association between BMI milestones and K-CPT outcomes were sex-specific. In males, older age and a higher magnitude of BMI rebound were associated with fewer OE, potentially reflecting greater efficiency in sustaining attention. Conversely, males with earlier BMI peak and rebound exhibited greater variability in response speed and higher OE. These patterns potentially reflect less stable attentional efficiency and greater difficulty sustaining attention and detecting targeted stimuli throughout the task. Additionally, an early peak-rebound pattern was associated with higher PR errors relative to the normative sample, potentially suggesting difficulty disengaging from prior response patterns. An early-peak, late-rebound pattern showed elevated responding to nontarget stimuli and potentially reflecting greater impulsivity, despite exhibiting slower overall response rates. In contrast, an early-peak, normal-rebound pattern was characterized by more stable, sustained attention and no observable difficulties maintaining task engagement.

Among females, patterns were less pronounced. Higher BMI at peak was associated with greater response-speed variability, while an early-peak-rebound pattern corresponded to better inhibitory control but lower attentional efficiency, potentially reflecting a more conservative performance style prioritizing accuracy over speed. Prior research suggests that associations between adiposity and EF, including inhibitory control, may vary by sex, although the direction and magnitude of these differences have not been consistent across studies.^46,47,48^ The limited associations in females may be attributable to sex-specific differences in hormonal regulation of growth and brain maturation,^49,50,51,52^ including prefrontal–striatal networks that support inhibition and attention. Our exploratory sex-specific findings support further longitudinal investigation of BMI-cognition associations through a sex-specific lens and suggest that early-life adiposity growth patterns may have differential implications for inhibitory regulation in males than in females.

Our broader findings for the overall sample align with existing obesity-neurobehavioral research,^53,54,55,56^ which has reported associations between children’s high BMI and maladaptive EF dimensions, including inhibition and attention.^42,54,55,56,57,58^ Children with obesity are prone to impulsive eating, possibly exacerbating difficulties in self-regulation,^59,60,61^ which has also been associated with poorer academic performance.^62,63^ However, these relationships may reflect shared genetic, environmental, nutritional, and developmental factors rather than direct effects of adiposity on cognition. For example, dietary intake and eating patterns may contribute to the observed associations because they can influence both childhood BMI trajectories and neurobehavioral development. Eating is a brain-regulated behavior fundamental to childhood growth, and poor nutrition and unhealthy eating patterns have been associated with higher BMI and poorer inhibitory control.^64,65,66,67^ It is important to note that this relationship may also be bidirectional, as poorer inhibitory control may contribute to unhealthy eating patterns, excess caloric intake, and subsequent increases in BMI.^68,69^ Our findings should also be considered within the broader evidence suggesting that BMI and EF may codevelop across childhood. Prior studies have identified inverse relationships between BMI and EF trajectories, including subgroups characterized by higher BMI and poorer EF beginning early in childhood.^70,71^ These findings suggest that early differences in EF may precede or contribute to subsequent BMI trajectories rather than adiposity exclusively influencing later neurocognitive function. Altogether, our observed associations may, in part, reflect bidirectional or shared developmental processes linking early growth and neurobehavioral regulation. Nevertheless, because no studies to our knowledge have specifically examined BMI peak and rebound in relation to later inhibitory control and attention, our study provides preliminary evidence that BMI trajectory milestones may relate to sensitive neurocognitive domains, highlighting potential developmental windows for targeted obesity and behavioral interventions.

### Strengths and Limitations

Strengths of our study include its prospective design and multiple measures of early-life growth and inhibitory control outcomes obtained by highly trained staff using standardized protocols. Limitations include estimating BMI peak and rebound from statistical models rather than direct observation and visual inspection of child-specific BMI-for-age curves^33^; however, visual inspection can be highly variable between observers and introduce measurement errors, and modeling the entire BMI trajectory (birth to childhood) may yield more precise milestone estimates.^40^ Our derived peak-rebound categories within our cohort may not generalize to other populations. Testing multiple K-CPT outcomes and BMI milestone parameters increased the risk of false-positive results. However, because we chose not to adjust for multiple comparisons, these findings should be considered exploratory and interpreted based on the coherence and consistency of associations across related measures.^72,73^ Because K-CPT outcomes were predominantly available at the 4 to 5–year assessment, with substantially fewer observations at ages 6 to 7 years, the repeated-measures estimates may have been more strongly influenced by the earlier assessment, despite linear mixed-effects models accommodating unbalanced data. Moreover, although the K-CPT is designed and normed for children aged 4 to 7 years, individual measures may exhibit different sensitivity across developmental stages, and performance may be influenced by factors such as age, attention, and task engagement. Furthermore, evidence supporting the psychometric properties of individual K-CPT indices in young children remains limited. The observed associations were generally modest in magnitude, which limits the strength of the conclusions and warrants cautious interpretation and replication in future studies. Small sample size, particularly for BMI peak-rebound categorical groups, may have limited precision. Additionally, we did not account for maternal or early-life adversity, which may represent an important potential confounder given its associations with both higher BMI trajectories and poorer neurocognitive outcomes. We were unable to account for dietary intake and eating patterns, which may represent important residual confounders given their potential relationships with both childhood BMI trajectories and neurocognitive development. Additionally, these findings may not generalize to children in other settings.

## Conclusions

In this cohort study, we provide preliminary evidence that early-life BMI peak-rebound patterns across childhood may be associated with aspects of cognitive impairments, specifically in inhibitory control, extending the literature beyond the well-established effects of metabolic conditions. Understanding whether early BMI growth patterns in childhood coincide with differences in inhibitory control and attention may help identify developmental periods when nutritional, behavioral, and healthy-growth interventions could be most relevant. Because BMI developmental milestones are derived from routinely collected growth measures, further research could determine whether these patterns provide useful early indicators of children who may benefit from additional developmental or behavioral support. Importantly, these associations should be understood within the broader social, nutritional, and environmental contexts that shape both childhood growth and neurobehavioral development rather than as characteristics inherent to the population studied. While preliminary, our results suggest that tracking children’s BMI trajectories across childhood may help identify children at risk not only for obesity but also for EF problems, potentially enabling earlier interventions to promote healthy development.

## Notes

Supplement 1. eMethods. eTable 1. Sex-Specific Associations of Ages at Body Mass Index (BMI) Peak and Rebound, and Magnitudes of BMI at Peak and Rebound With K-CPT T-Scores eTable 2. Association Between Timing of BMI Peak-Rebound Patterns and K-CPT T-Scores in Male Children (n=278) eTable 3. Association Between Timing of BMI Peak-Rebound Patterns and K-CPT T-Scores in Female Children (n=261)

Supplement 2. Data Sharing Statement
