# Supplementary material for: Body Mass Index Peak-Rebound Patterns and Inhibitory Control Among Young Children
Source: JAMA Netw Open. 2026 Nov 13;9(11):e2642834. doi: 10.1001/jamanetworkopen.2026.42834 (PMC13768952; doi:10.1001/jamanetworkopen.2026.42834)
Supplement: Supplementary file 1 [file jamanetwopen-e2642834-s001.pdf]

Supplemental Online Content

Lane JM, Aris IM, Hsu HHL, et al. Body mass index peak-rebound patterns and inhibitory control among young children. *JAMA Netw Open*. 2026;9(11):e2642834. doi:10.1001/jamanetworkopen.2026.42834

eMethods.
eTable 1. Sex-Specific Associations of Ages at Body Mass Index (BMI) Peak and Rebound, and Magnitudes of BMI at Peak and Rebound With K-CPT T-Scores
eTable 2. Association Between Timing of BMI Peak-Rebound Patterns and K-CPT T-Scores in Male Children (n=278)
eTable 3. Association Between Timing of BMI Peak-Rebound Patterns and K-CPT T-Scores in Female Children (n=261)

This supplemental material has been provided by the authors to give readers additional information about their work.

© 2026 Lane JM et al. *JAMA Network Open.*

eMethods.

Exposure: BMI Milestone Estimates

To estimate BMI milestones (i.e., age and magnitude of peak and rebound), we used statistical models, rather than direct observations and visual inspection of individual BMI-for-age curves, which are prone to large inter-observer variation [32]. In line with prior work, we fitted child-specific BMI curves from birth to 8 years using mixed-effects models with natural cubic spline functions for age, with 4 knots placed at equally spaced percentiles of the age distribution to capture the non-linear trend in BMI [39, 40]. We derived sex-specific trajectories by including interactions of child sex with spline terms as fixed effects in the model. We also included random effects for the intercept, linear age slopes, and spline functions to account for repeated BMI measures in the same child. Using the fitted model, we estimated the exact age (in years) at peak in infancy and rebound in childhood by differentiating each child-specific BMI curve. It is important to note that although we used BMI from birth to 8 years to derive child-specific BMI curves, our exposure of interest pertains only to the specific BMI milestones (i.e., BMI peak in infancy and rebound in childhood) which are point measures on the BMI curve that precede the outcome. Specifically, the peak and rebound are located at ages where the derivative of the curve equals to zero i.e., at the maximum inflection point during infancy for peak, and the minimum inflection point during childhood for rebound. We estimated the magnitude (in kg/m2) of BMI at peak and rebound as the highest and lowest points, respectively, of each child-specific BMI curve.

Outcome: Inhibitory Control: *KCPT Task Description*

The K-CPT takes approximately 7.5 minutes to administer, making it both highly suitable and low-burden for younger children. The K-CPT tasks included omission errors, commission errors, hit reaction time (HRT), and its standard error (HRT SE), perseveration responses, and response style. Incorrect responses were categorized as omission errors (failure to respond when required) and commission errors (responding when not required), with higher scores indicating greater error rates. HRT and HRT SE capture response speed and response-time variability across the test. Higher scores reflect unusually slow reaction times and greater variability, respectively, both of which indicate reduced attentiveness. Perseverations, responses made within 100 ms of a stimulus, were reflected in random, repetitive, or anticipatory responses, with higher scores indicating a greater frequency of perseverative errors. Based on these measures, the K-CPT can characterize a child’s response style—a signal detection metric that reflects their natural tendency toward a liberal, conservative, or balanced approach when managing the speed–accuracy trade-off. Higher response style scores reflect a more conservative approach, preferring accuracy over speed, lower scores indicate a more liberal approach, prioritizing speed at the risk of errors, and midrange scores suggest a balanced speed–accuracy trade-off. The K-CPT (Second Edition; MHS) scoring software automatically generates T-scores and percentiles for each performance indicator using the test’s age- and sex-specific normative dataset. For this study, we used the standardized K-CPT index T-scores as the primary outcomes. T-scores are calculated relative to the validated K-CPT norms, where a value of 50 represents the mean for children of the same age and sex, and the standard deviation is 10. Higher T-scores (≥1 SD above the mean) indicate poorer performance, suggesting greater risk for sustained attention and inhibitory control-related difficulties. No additional inputs beyond the child’s age and sex, as required by the scoring system, were used in generating these standardized scores.

Covariates

Trained research staff collected information on the child's sex, gestational age at delivery, birthweight, age at neurobehavioral assessment, maternal intelligence quotient (IQ; estimated using the Wechsler Abbreviated Scale of Intelligence), maternal BMI (calculate using the measured weights obtained in the 2nd trimester), and secondhand smoke exposure via structured, in-person interviews conducted with mothers at the research site located within the Mexican National Institute of Perinatology. Socioeconomic status (SES) was determined using an index developed by the Asociación Mexicana de Agencias de Investigación de Mercados y Opinión Pública (AMAI), which incorporates 13 indicators from the prenatal questionnaire to generate a composite SES score into 6 categories. Based on this index, we categorized participants into low, medium, or high SES groups relative to the distribution within our study population.

© 2026 Lane JM et al. *JAMA Network Open.*

eTable 1. Sex-Specific Associations of Ages at Body Mass Index (BMI) Peak and Rebound, and Magnitudes of BMI at Peak and Rebound With K-CPT T-Scores

	Commisions T-score[a]			Hit RT T-score[a]			Hit RT Standard Error T-Score[a]		
Predictors	**Male (N=277)**	**Female (N=261)**	**P-interaction**	**Male (N=277)**	**Female (N=261)**	**P-interaction**	**Male (N=277)**	**Female (N=261)**	**P-interaction**
Age at BMI peak	-0.66 (-2.58, 1.26)	1.23 (-0.38, 2.84)	0.14	-0.69 (-2.76, 1.38)	0.03 (-1.63, 1.70)	0.42	-1.38 (-2.97, 0.22)	0.79 (-0.58, 2.16)	0.03
BMI at peak	0.65 (-0.49, 1.80)	0.61 (-0.58, 1.80)	0.87	0.06 (-1.17, 1.29)	0.54 (-0.69, 1.77)	0.52	0.58 (-0.37, 1.53)	0.98 (-0.03, 2.00)	0.49
Age at BMI rebound[b]	0.64 (-2.54, 3.82)	-1.70 (-5.00, 1.59)	0.40	-0.36 (-3.79, 3.08)	**4.49 (1.07, 7.90)**	0.10	-1.44 (-4.10, 1.21)	1.58 (-1.24, 4.40)	0.18
BMI at rebound[b]	1.61 (-2.41, 5.64)	-2.76 (-7.28, 1.76)	0.28	-0.94 (-5.29, 3.41)	4.64 (-0.01, 9.30)	0.16	-1.71 (-5.08, 1.66)	1.51 (-2.35, 5.37)	0.24
	**Omissions T-score[a]**			**Perseverations T-score[a]**			**Response Style T-score[a]**		
Predictors	**Male (N=277)**	**Female (N=261)**	**P-interaction**	**Male (N=277)**	**Female (N=261)**	**P-interaction**	**Male (N=277)**	**Female (N=261)**	**P-interaction**
Age at BMI peak	**-2.55 (-4.42, -0.68)**	0.04 (-1.40, 1.47)	0.04	-2.08 (-6.09, 1.92)	1.56 (-1.35, 4.47)	0.11	0.01 (-2.22, 2.23)	-0.95 (-2.77, 0.86)	0.37
BMI at peak	0.57 (-0.56, 1.69)	0.67 (-0.39, 1.73)	0.92	0.14 (-2.23, 2.51)	1.01 (-1.13, 3.16)	0.45	-0.31 (-1.62, 1.00)	-0.67 (-2.01, 0.67)	0.71
Age at BMI rebound[b]	**-3.20 (-6.31, -0.09)**	0.07 (-2.86, 3.00)	0.15	-2.77 (-9.38, 3.83)	1.08 (-4.86, 7.02)	0.37	-1.46 (-5.11, 2.18)	-0.06 (-3.67, 3.55)	0.44
BMI at rebound[b]	**-4.30 (-8.24, -0.37)**	0.83 (-3.19, 4.86)	0.08	-1.10 (-9.47, 7.28)	-0.29 (-8.44, 7.86)	0.67	-3.17 (-7.80, 1.46)	2.39 (-2.59, 7.38)	0.08

[a] Adjusted for SES index, birth weight, gestational age at delivery, secondhand smoke exposure, maternal BMI, and maternal IQ.
[b] Additionally adjusted for age and magnitude of BMI at peak.

© 2026 Lane JM et al. *JAMA Network Open.*

eTable 2. Association Between Timing of BMI Peak-Rebound Patterns and K-CPT T-Scores in Male Children (n=278).

	**T-score Outcomes**					
Predictors	**Commissions**	**Hit RT**	**Hit RT Standard Error**	**Omissions**	**Perseverations**	**Response Style**
Normal AP + Normal AR	reference	reference	reference	reference	reference	reference
Early AP + Early AR	3.57 (-0.57, 7.71)	-2.03 (-6.50, 2.43)	2.57 (-0.89, 6.03)	2.49 (-1.62, 6.61)	**9.51 (0.99, 18.03)**	-4.15 (-8.82, 0.52)
Early AP + Normal AR	0.58 (-3.15, 4.30)	-3.08 (-7.11, 0.94)	-2.44 (-5.56, 0.67)	**-3.93 (-7.61, -0.25)**	-1.32 (-9.03, 6.39)	-3.10 (-7.33, 1.14)
Early AP + Late AR	**3.83 (0.05, 7.61)**	**-4.99 (-9.07, -0.90)**	0.60 (-2.56, 3.76)	-0.66 (-4.41, 3.08)	3.17 (-4.65, 10.99)	-3.77 (-8.07, 0.52)
Normal AP + Early AR	1.80 (-1.65, 5.24)	-2.29 (-6.01, 1.43)	0.02 (-2.86, 2.90)	-0.78 (-4.20, 2.64)	1.93 (-5.17, 9.04)	**-3.98 (-7.87, -0.08)**
Normal AP + Late AR	2.15 (-0.88, 5.19)	-0.43 (-3.71, 2.86)	0.66 (-1.87, 3.20)	-0.67 (-3.64, 2.31)	4.02 (-2.29, 10.34)	-2.97 (-6.45, 0.51)
Late AP + Early AR	0.00 (-7.11, 7.12)	-4.86 (-12.55, 2.83)	**-6.72 (-12.66, -0.78)**	**-8.13 (-15.16, -1.11)**	-4.39 (-19.11, 10.34)	-6.74 (-14.83, 1.36)
Late AP + Normal AR	2.89 (-0.93, 6.71)	-3.10 (-7.19, 0.99)	-1.04 (-4.18, 2.10)	**-4.37 (-8.03, -0.70)**	6.25 (-1.65, 14.16)	-3.55 (-7.92, 0.83)
Late AP + Late AR	-3.17 (-8.30, 1.95)	-0.99 (-6.52, 4.54)	-0.86 (-5.15, 3.42)	0.39 (-4.70, 5.48)	-3.65 (-14.20, 6.90)	2.96 (-2.82, 8.75)

AP: Age at BMI peak; AR: Age at BMI rebound. Early AP or AR = age≤25th percentile; Normal AP or AR = age >25th or ≤75th percentile. Late AP or AR = age >75th percentile.
All models adjusted for age at outcome measurement, SES index, birth weight, gestational age at delivery, secondhand smoke exposure, maternal BMI, and maternal IQ.

© 2026 Lane JM et al. *JAMA Network Open.*

eTable 3. Association Between Timing of BMI Peak-Rebound Patterns and K-CPT T-Scores in Female Children (n=261).

	T-score Outcomes					
Predictors	**Commissions**	**Hit RT**	**Hit RT Standard Error**	**Omissions**	**Perseverations**	**Response Style**
Normal AP + Normal AR	reference	reference	reference	reference	reference	reference
Early AP + Early AR	**-5.74<br>(-11.14, -0.35)**	1.42<br>(-4.19, 7.03)	1.27<br>(-3.38, 5.91)	**5.25<br>(0.46, 10.04)**	-7.70<br>(-17.45, 2.04)	**6.80<br>(0.83, 12.77)**
Early AP + Normal AR	-0.24<br>(-5.39, 4.92)	0.38<br>(-4.95, 5.72)	1.52<br>(-2.91, 5.95)	**4.98<br>(0.39, 9.56)**	-1.37<br>(-10.69, 7.96)	1.88<br>(-3.87, 7.63)
Early AP + Late AR	-2.14<br>(-6.95, 2.68)	-2.87<br>(-7.86, 2.12)	-1.76<br>(-5.91, 2.38)	0.76<br>(-3.52, 5.05)	-0.92<br>(-9.63, 7.80)	-1.15<br>(-6.51, 4.22)
Normal AP + Early AR	-1.60<br>(-5.82, 2.63)	-1.84<br>(-6.20, 2.51)	-2.24<br>(-5.87, 1.39)	0.49<br>(-3.28, 4.26)	-2.79<br>(-10.46, 4.87)	3.83<br>(-0.92, 8.59)
Normal AP + Late AR	-2.81<br>(-6.41, 0.78)	1.46<br>(-2.25, 5.18)	0.09<br>(-2.99, 3.18)	1.40<br>(-1.80, 4.60)	-3.25<br>(-9.75, 3.25)	2.02<br>(-2.00, 6.04)
Late AP + Early AR	-1.31<br>(-7.38, 4.75)	0.55<br>(-5.66, 6.75)	2.32<br>(-2.88, 7.53)	4.51<br>(-0.91, 9.93)	-3.26<br>(-14.28, 7.75)	-1.04<br>(-7.93, 5.85)
Late AP + Normal AR	-1.50<br>(-5.46, 2.45)	-1.23<br>(-5.31, 2.85)	0.29<br>(-3.10, 3.69)	1.00<br>(-2.52, 4.52)	3.42<br>(-3.74, 10.58)	-0.58<br>(-5.00, 3.84)
Late AP + Late AR	2.69<br>(-2.70, 8.08)	5.02<br>(-0.56, 10.61)	3.36<br>(-1.27, 8.00)	2.57<br>(-2.22, 7.37)	-1.39<br>(-11.14, 8.35)	-1.73<br>(-7.73, 4.26)

AP: Age at BMI peak; AR: Age at BMI rebound. Early AP or AR = age≤25th percentile; Normal AP or AR = age >25th or ≤75th percentile. Late AP or AR = age >75th percentile.
All models adjusted for age at outcome measurement, SES index, birth weight, gestational age at delivery, secondhand smoke exposure, maternal BMI, and maternal IQ.

© 2026 Lane JM et al. *JAMA Network Open.*
