# Supplementary material for: Body Mass Index Peak-Rebound Patterns and Inhibitory Control Among Young Children
Source: JAMA Netw Open. 2026 Nov 13;9(11):e2642834. doi: 10.1001/jamanetworkopen.2026.42834 (PMC13768952; doi:10.1001/jamanetworkopen.2026.42834)
Supplement: Supplementary file 2 [file jamanetwopen-e2642834-s002.pdf]

Data Sharing Statement

Lane. Body Mass Index Peak-Rebound Patterns and Inhibitory Control Among Young Children. *JAMA Netw Open*. Published November 06, 2026. doi:10.1001/jamanetworkopen.2026.42834

Data
Data available: No

Additional Information
Explanation for why data not available: All data are available upon request to robert.wright@mssm.edu.
